# Effectivity of a mHealth intervention for individuals with obesity: a study protocol for a controlled intervention study

**DOI:** 10.1186/s13102-021-00337-6

**Published:** 2021-09-17

**Authors:** Julian Fritsch, Katharina Feil, Susanne Weyland, Detlef Schmidt, Darko Jekauc

**Affiliations:** Institute for Sport and Sport Science, Karlsruhe Institute of Technology, Engler-Bunte-Ring 15, 76131 Karlsruhe, Germany

**Keywords:** mHealth, Obesity, Effectivity, Physical activity, Implicit processes, Explicit processes

## Abstract

**Background:**

Obesity is considered an epidemic problem with an increasing number of individuals affected. The physical and psychological complaints associated with obesity point to the importance of implementing effective interventions. Innovative mHealth applications appear to be promising in helping provide a continuous and flexible support during the intervention. Since research on mHealth interventions is still relatively sparse, the main goal of the current study is to assess the effectiveness of an mHealth obesity intervention in terms of weight reduction, health behaviours as well as health-related quality of life. In addition, the study aims to investigate various psychological explicit and implicit processes associated with physical activity behaviour.

**Methods:**

The study includes quantitative and qualitative methods. Regarding the quantitative methods, the goal is to recruit up to 450 individuals at baseline in different obesity centres across Germany with some of these centres offering an mHealth intervention. All individuals who agree to take part in the mHealth intervention will be assigned to the intervention group, while all other individuals will be assigned to the control group. The mHealth obesity intervention consists of three stays at an obesity centre, with approximately six months between stays during which patients are supported by the digital platform CASPAR. The study includes three measurements with a baseline measurement and two follow-up measurements, one after six months and one after twelve months. To assess the effectiveness of the intervention, body weight, physical activity behaviour, eating behaviour as well as health related quality of life will be assessed. In addition, motivation, intentions, self-efficacy, enjoyment, and habit will be used to assess the psychological processes related with physical activity behaviour. A multivariate analysis of variance with repeated measurement and latent growth curve models will be used to compare the development of the variables within the two groups. In relation to the qualitative methods, interviews with individuals of the intervention group will be conducted to shed light on the applicability, acceptance, and usability of the mHealth intervention.

**Discussion:**

This study may provide a valuable insight into the potential of mHealth obesity interventions and the psychological processes related to physical activity behaviour.

*Trial registration* The trial has been registered with the German Register of Clinical Studies (DRKS) on June 30, 2021 under the registration number: DRKS00024836.

## Background

Obesity is considered an epidemic problem, with most European countries projected to have an obesity prevalence of at least 20% by the year 2025 [1]. For instance, in Germany, where the present study will be conducted, the results of epidemiological studies indicate a prevalence rate between 18 and 23% in the adult population [2, 3]. Obesity is associated with a higher risk of physical complaints, such as cardiovascular diseases [4] or type 2 diabetes mellitus [5], as well as mental complaints, such as depression [6] or anxiety disorders [7]. The complaints are not only a burden for the individual, but are also associated with substantial health care costs for the society [8]. Given the health and economic burden associated with obesity, the World Health Organization (WHO) has set the target to halt obesity prevalence at 2010 levels by the year 2025 [9]. However, epidemiological studies indicate that this target is unlikely to be achieved globally [10] as well as in Germany specifically [2].

The trends in the prevalence of obesity underscore the importance of implementing effective obesity interventions. In this regard, the increasing usage of mobile health (mHealth) interventions appears promising in order to reduce sedentary behaviour and to enhance physical activity and healthy eating [11]. mHealth interventions can facilitate access to health care services for a larger portion of the population, provide flexible and rapid feedback to individuals, and tailor interventions to the individuals’ needs [12]. For instance, mHealth interventions can make it easier for individuals to do their exercises in places and at times that are convenient for them. This option can be particularly beneficial for individuals with obesity who often feel uncomfortable in regular fitness settings [13]. However, despite these promising opportunities of mHealth interventions, it is also important to acknowledge their potential challenges. There are several issues, such as doubts about data security, technical difficulties with the software or lack of social support, which may cause individuals to disengage from the intervention [12].

Obesity often results from an imbalance between too little energy expenditure and too much energy intake [14]. Therefore, to achieve weight loss as a primary goal, obesity interventions should help individuals be more physically active and eat healthier. Moreover, various studies indicate that obesity is negatively associated with indicators of health-related quality of life [15]. Individuals with obesity often report that because of their weight they experience physical complaints, have problems during their daily activities, or have difficulties to move in public [16]. In the same vein, individuals with obesity report often experiencing negative affective states, such as anger, fear or shame, in their daily life [17]. Because indicators of health-related quality of life are associated with the use of health services [18] as well as mortality [19], they are also important indicators of the success of an obesity intervention.

To increase the effectiveness of obesity interventions it is further important to understand the psychological processes that are associated with behaviour change [20]. In this study, we focus on the psychological processes related to physical activity. In this regard, a large number of studies shows that individuals who have a more autonomous motivation are more likely to be physically active [21]. In addition, dual-process theories that attempt to explain physical activity behaviour emphasize the role of both more explicit as well as more implicit psychological processes [22, 23]. On the one hand, individuals’ intention, self-efficacy, and enjoyment towards physical activity as more explicit processes have been consistently associated with physical activity [24–26]. On the other hand, habit is considered a more implicit process that has also been shown to be associated with physical activity [27].

To summarize, the high prevalence of obesity is a major problem from both an individual and societal perspective [1]. While mHealth interventions may provide new promising ways to combat obesity, scientific evidence of their effectiveness is still sparse [11]. Therefore, the primary purpose of this study is to evaluate the effectiveness of an mHealth obesity intervention by assessing body weight as well as indicators of physical activity, eating behaviours and health-related quality of life. In addition, the secondary purpose of this study is to assess more explicit (i.e., motivation, intention, self-efficacy, enjoyment) and more implicit (i.e., habit) psychological processes associated with physical activity behaviour change through the use of mixed methods.

### Methods

#### Sampling and participants

The patients are recruited from obesity centres across Germany. Currently, the participants will be recruited from seven obesity centres and it is possible that more obesity centres will participate. Of the seven obesity centres, four offer an mHealth intervention and three do not. The inclusion criteria are that patients are at least 18 years old, have a basic knowledge of the German language, and a BMI ≥ 30. During their first stay at the obesity centre, patients will be asked to participate in the study. Due to the nature of the study, neither participants nor staff can be blinded to allocation. Thus, patients attending an obesity centre that offers an mHealth intervention will be asked whether they want to take part in the mHealth intervention. The patients who agree to take part in such an intervention will be assigned to the intervention group, while those who do not want to take part in such an intervention will be assigned to the control group. Moreover, patients attending a obesity centre that does not offer mHealth interventions will be assigned to the control group. The patients will receive no financial compensation for their participation in the study.

Given the insufficient evidence for the effectiveness of mHealth obesity interventions [28], a small effect size was considered in the power analysis. Conservatively estimating the effect size by *η*^2^ = 0.01, *α* = 0.05, 1-*β* = 0.80, with two groups, a correlation on among repeated measures of *r* = .30 and a design with three repeated measurement occasions, using an ANOVA with repeated measurements (within-between interaction), the calculated total sample size is 225 participants, nearly equally distributed over two groups. Since we expect a dropout rate of about 50% [29], the plan is to recruit 450 patients.

#### mHealth obesity intervention

The obesity intervention consists of three stays at an obesity centre with about six months between the stays. The first stay lasts three weeks and the two following stays last one week each. The focus for the time at the obesity centres lies on nutritional therapy and exercise therapy, delivered in both individual and group sessions. The stays at the obesity centres are organized by groups. Each new group consists of a maximum of twelve participants. It is intended that a group also stays together for the second and third stay at the obesity centre. However, depending on the patients’ availability, this is not always possible. Moreover, one criterion for another stay is that patients have not gained weight compared to their last stay.

Patients can choose to have a digital supervision during the time between the stays (i.e., patients who do not choose that option can be assigned to the control group). This supervision is applied through the use of the digital platform CASPAR. CASPAR has been developed by the German company “Goreha” and offers exercises as well as seminars to be used digitally (for more information see: https://www.caspar-health.com/en). With relevance for obesity, CASPAR consists of physical exercises, nutritional advices as well as psychological content (e.g., progressive muscle relaxation). Patients will be introduced to CASPAR while still at the obesity centre, and will receive an individualized training plan when they leave the centre. Between each stay, patients are supposed to spend up to 90 min a week with CASPAR for a total of 24 weeks (meaning 2160 min in total). It is up to the patients how they accumulate these minutes (they could do 150 min in one week and 30 min in another week). Only when patients are not active in CASPAR for at least 90 min in six subsequent weeks, the supervision through CASPAR is cancelled. If patients are inactive, they can be encouraged through CASPAR to be active again. Moreover, patients are continuously supervised by qualified therapists. Depending on the obesity centre, this supervision is either done by therapists employed by the provider of CASPAR or by therapists working at the obesity centre. During the intervention, patients can ask the therapists to adapt the exercises when needed. In addition, patients can record themselves when doing the exercises and ask for feedback. In case of possible harm, patients can be referred to appropriate treatment. Moreover, all patients can receive additional therapies depending on their health status.

#### Control group

Patients who do not want to take part in the mHealth intervention will be assigned to the control group. In addition, there are three other obesity centres that do not offer an mHealth intervention from which patients will be recruited. In these centres, patients have their first stay in an obesity centre organized by groups with about twelve patients. During this stay, there are individual and group sessions with a focus on nutritional therapy and exercise therapy. When leaving the obesity centre, the patients will receive an exercise plan and dietary recommendations that should help them implement what they have learnt at the obesity centre. Equal to the intervention groups, all patients can receive additional therapies depending on their health status.

It is important to note that for logistical reasons, the involved obesity centres have differences regarding the type of intervention. While in two of the obesity centres the first stay of the patients lasts three weeks, in one centre the first stay lasts one week. In addition, two of the obesity centres offer two additional stays, while one offers no additional stay.

#### Data collection

This study consists of quantitative as well as qualitative data collection methods.

##### Quantitative data collection methods

**Fig. 1:**
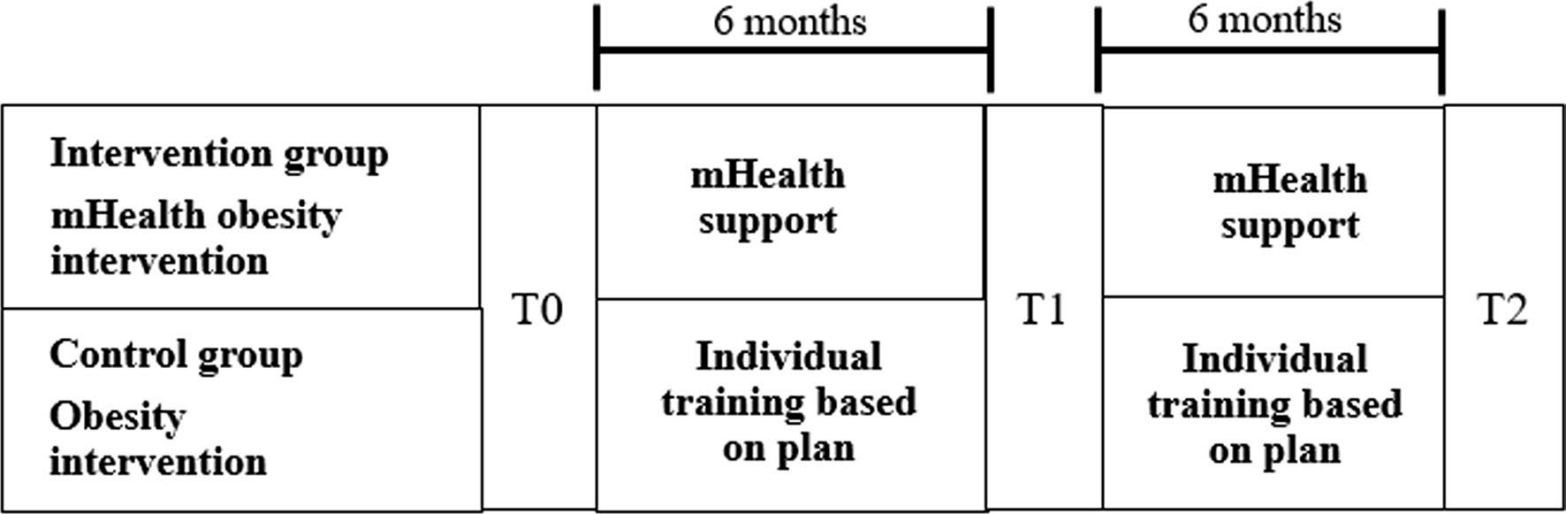
Study design

The data collection will start in July 2021 and the recruitment of patients for the baseline measurement is planned to finish in May 2022. The data collection will include three measurements (see Fig. 1). For all patients, the baseline measurement will take place during the first stay at the obesity centre. The follow-up measurements are aligned with the patients’ stays in the intervention group. For patients from obesity centres who offer an mHealth intervention, the first follow-up measurement will take place during the second stay at an obesity centre and the second follow-up measurement during the third stay. Patients recruited from obesity centres that do not offer an mHealth intervention will have the first follow-up measurement after six and the second follow-up measurement after twelve months, with the questionnaires directly sent to them. Questionnaires will also be sent directly to patients who are in obesity centres that offer an mHealth intervention (and thus potentially include further stays at the obesity centre), but do not attend them (e.g., due to weight gain, another stay was not approved by the insurance provider). Depending on the patients’ preference, the questionnaires will be sent either by e-mail or by post and a reminder will be sent if the patients do not return the questionnaires within two weeks.

**Fig. 2:**
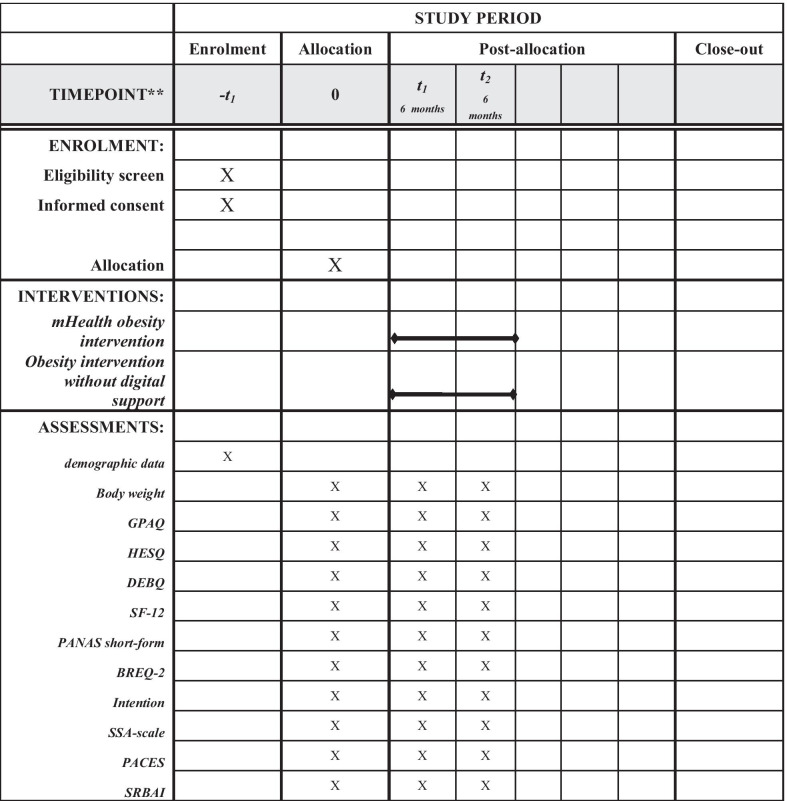
SPIRIT timetable

Demographic information, including age, gender and current employment situation, will be asked at baseline. All other instruments described below will be used at the baseline measurement and the two follow-up measurements. The SPIRIT timetable of the study is presented in Fig. 2.

#### Body weight

The body weight will be provided by the obesity centres if the patients agree that this information can be forwarded. In case the patients are not at the obesity centre during the measurement, the patients will be asked to report their weight themselves. The patients will be asked to wear light clothing and no shoes during the measurement and to report their weight to the nearest 0.1 kg.

#### Global physical activity questionnaire

Physical activity will be assessed with the Global Physical Activity Questionnaire (GPAQ) [30]. The GPAQ consists of 16 items measuring volume and intensity of moderate and vigorous intensity in the areas of work, transport, and discretionary time. Moreover, the GPAQ measures daily sedentary time. The GPAQ has been shown to have a 10-days test-retest reliability of *r* = .83 to *r* = .96 and a 3-months test-retest-reliability of *r* = .53 to *r* = .83 [31]. The GPAQ also shows a moderate agreement with accelerometer measures [32].

#### Healthy eating style

Healthy eating style will be measured with the 12-item questionnaire (HESQ) assessing food consumption patterns [33]. The questionnaire has a seven-point Likert scale from strongly disagree (1) to strongly agree (7). In a previous study, this scale had an acceptable reliability of Cronbach᾽s α = 0.77 and showed an adequate one-factor solution [33].

#### Dutch eating behaviour questionnaire

The Dutch Eating Behaviour Questionnaire (DEBQ) will be used to assess different eating styles associated with obesity [34]. The DEBQ consists of 30 items, including three different subscales: restraint eating, emotional eating, and external eating. Each subscale has 10 items and the responses are given on a five-point Likert scale from never (1) to very often (5). A previous study has shown Cronbach᾽s α = 0.92 for restraint eating, α = 0.94 for emotional eating and α = 0.89 for external eating [34]. In addition, scores in both the emotional eating and external eating subscales have been shown to be higher in individuals with obesity [34]. For this reason, only these two subscales will be used in the present study.

#### SF-12

The SF-12 will be used to measure health-related quality of life [35]. The SF-12 consists of 12 items with continuous and dichotomous response formats, which comprise the two dimensions physical and mental health. Studies have shown Cronbach᾽s α = 0.87 for mental health and α = 0.83 for physical health [35]. The SF-12 was shown to be applicable independent of the current health status [36] and to be associated with other indicators of physical and mental health [37].

#### Positive and negative affect schedule

The Positive and Negative Affect Schedule (PANAS) short form [38] will be used to measure emotional well-being as one aspect of health-related quality of life. This questionnaire consists of the two subscales positive affect and negative affect, each measured by five items. The patients will be asked to what extent they generally feel these affective states on a five-point Likert scale, ranging from not at all (1) to extremely (5). The 8-weeks test-retest reliability of the short-form was *r* = .84 [38]. The positive affect subscale was shown to have a positive association with subjective well-being, and the negative affect subscale was shown to have a negative association with subjective well-being [38].

#### Behavioural regulation exercise questionnaire-2

The Behavioural Regulation Exercise Questionnaire-2 (BREQ-2) [39] will be used to measure motivational regulation. The questionnaire consists of 19 items with a five-point Likert scale, ranging from not true at all (1) to very true (5). The BREQ-2 measures five dimensions of motivation: intrinsic motivation, identified regulation, introjected regulation, extrinsic motivation, and amotivation. The reliability has been shown to range from acceptable to good (α = 0.73 − 0.86) for the different dimensions [39]. In addition, the subscales representing more autonomous types of motivation were shown to be positively associated with health behaviours [40].

#### Intention

Intention will be assessed with a two-items questionnaire, namely whether the individuals will intend and whether they will be sure to be physically active [41]. The answers will be given on a seven-point Likert scale, ranging from totally disagree (1) to totally agree (7). The Cronbach’s α of this scale was at 0.83 in a previous study [41].

#### Self-efficacy

Self-efficacy will be assessed with a questionnaire specifically related to self-efficacy towards physical activity (SSA-scale) [42]. The questionnaire consists of twelve items with a five-point Likert scale, ranging from not sure at all (1) to very sure (5). The questionnaire has a Cronbach’s α of 0.89 and was shown to distinguish between physically active and inactive individuals [42].

#### PACES

An adapted short-form of the Physical Activity Enjoyment Scale (PACES) will be used to assess the individuals’ enjoyment in relation to physical activity [43]. In this study, we will use those four items that focus on the subjective experience of enjoyment (e.g., “I find physical activity pleasurable”). The PACES has been shown to be related to the physical activity levels of individuals [43].

#### Habit

Habit will be assessed with the Self-Report Behavioural Automaticity Index (SRBAI) [44]. The SRBAI consists of four items with a seven-point Likert scale, ranging from strongly disagree (1) to strongly agree (5). Cronbach’s α has been shown to be between 0.86 and 0.88 [45]. Moreover, the SRBAI has been shown to have a positive association with physical activity [44].

#### Data collection of qualitative study

Semi-structured interviews will be conducted with ten to fifteen patients of the mHealth obesity intervention group. A purposive sampling [46] will be used to recruit (a) patients who are still active in the mHealth obesity intervention and (b) patients who had dropped out of this intervention. When providing informed consent for the study, patients are additionally asked if they would also like to be interviewed. Because patients should have sufficient experience with CASPAR for the interview, patients who agreed to the interview will be contacted after the second measurement (i.e., after they have had approximately six months of experience with CASPAR). Interview guidelines will be developed to target applicability, acceptance, and usability of the mHealth intervention. A specific focus will be on the emotional difficulties patients have in changing their behaviour. To ensure a natural flow of the interview, the order of the questions may vary depending on the patients’ responses [46]. In addition, patients will be encouraged to talk about personally relevant issues not directly related to the research questions. The interviews will last about 30 min and will be conducted via Skype or phone.

### Data analysis

#### Data analysis of quantitative data

All data related to personal information will be pseudo-anonymized. Data will be entered by a research assistant and checked independently for their accuracy by another research assistant. Multiple imputation or the full-information maximum likelihood approach will be employed to treat missing data [47]. We will conduct two analyses: (a) with all patients who agreed to participate in the intervention and (b) with only those patients who completed the intervention (i.e., they had three stays at an obesity centre). A multivariate analysis of variance with repeated measurements (2 groups x 3 time points) will be used to analyze the effects of the intervention on the dependent variables (i.e., body weight, physical activity, eating style & behaviour, health-related quality of life). For the analysis of the structure of determinants of physical activity (i.e., motivation, intention, self-efficacy, enjoyment, habit) and its interdependencies, structural equation modeling procedures will be applied. In particular, the development of the dependent variables will be analyzed using latent growth curve models [48]. Moreover, mediation analyses will be conducted to test whether the determinants of physical activity mediate the effects of the intervention on physical activity behaviour. The effects of age, gender, frequency of participation in the mHealth application (supplied by the provider), and the physical activity level outside of the intervention will be controlled.

#### Data analysis of qualitative data

After a verbatim transcription of the interviews, a reflexive thematic analysis approach will be used. Such an approach acknowledges that themes are actively generated by researchers resulting from their engagement with the data [49]. In particular, the analysis will follow the recursive six-phase model proposed by Braun and colleagues [50]. To ensure the rigour of the qualitative study, the data analysis will be carried out independently by two researchers. Moreover, other researchers in the team will act as critical friends helping consider alternative interpretations of the answers [51].

## Discussion

The objectives of this study are (1) to assess the efficacy of an mHealth obesity intervention in terms of weight reduction, health behaviours and health-related quality of life as well as (2) to examine the psychological processes related with physical activity behaviour. Regarding the second objective, psychological processes such as motivation, intention, self-efficacy, habit, and enjoyment will be examined. Moreover, interviews will be conducted to consider patient perspectives on the potential of mHealth interventions for behaviour change.

A look at the alarming trends in the prevalence of obesity [1] highlights the importance of implementing effective obesity interventions. In this regard, although mHealth interventions appear to offer innovative and flexible treatments tailored to the needs of the patients [12], more evidence is needed to assess their effectiveness [11]. In addition, it is important to keep the potential disadvantages in mind, such as technical problems with the software or the lack of social support [12]. One way to counteract these potential issues may be to provide ongoing supervision by a professional support team, as is the case with the mHealth intervention assessed in this study.

A strength of this study is the mix of different methods that may allow to understand the difficulties of individuals with obesity in their behaviour change as well as the potential of mHealth interventions to tackle these difficulties. Moreover, the longitudinal design of the study allows us to examine the trajectories in the outcome variables. On the contrary, a limitation of the study is the lack of randomization, which is not possible for ethical reasons (i.e., patients of the same obesity centre cannot be treated differently). Thus, because patients have the choice to participate in the mHealth intervention, potential selection bias could affect the results. In addition, differences in procedures across obesity centres may confound the effects of the mHealth obesity intervention itself. Finally, it is important to note that COVID-19 could affect the study. In particular, restrictions for obesity centres on patient admissions may impede recruitment of patients as well as their participation in subsequent visits.

To conclude, initial evidence points to the potential of mHealth interventions to promote health-related behaviours. Considering the need for long-term support for patients with obesity, mHealth interventions may hold particular promising for this population. However, because mHealth interventions are still in their infancy, more evidence is needed. Thus, the results of this study may make a valuable contribution to the existing body of knowledge on the effectiveness of such interventions in enhancing health-related indicators. Moreover, the results may also shed light on the importance of various psychological processes related to physical activity as a specific health-related behaviour in the context of mHealth obesity interventions.

## Data Availability

The datasets used and/or analysed during the current study are available from the corresponding author on reasonable request.

## Abbreviations

ANOVA: Analysis of variance
BREQ-2: Behavioural Regulation Exercise Questionnaire-2
DEBQ: Dutch Behaviour Eating Questionnaire
DRKS: German Register of Clinical Studies
GPAQ: Global Physical Activity Questionnaire
HESQ: Healthy Eating Style Questionnaire
mHealth: Mobile health
PACES: Physical Activity Enjoyment Scale
SF-12: Short Form 12
SSA-scale: Selbstwirksamkeit zur sportlichen Aktivitäts-Skala
SRBAI: Self-Report Behavioural Automaticity Index
WHO: World Health Organization
